# Incident Infarcts in Patients With Stroke and Cerebral Small Vessel Disease: Frequency and Relation to Clinical Outcomes

**DOI:** 10.1212/WNL.0000000000209750

**Published:** 2024-08-19

**Authors:** Una Clancy, Carmen Arteaga-Reyes, Daniela Jaime Garcia, Will Hewins, Rachel Locherty, Maria Del C. Valdés Hernández, Stewart J. Wiseman, Michael S. Stringer, Michael Thrippleton, Francesca M. Chappell, Angela C.C. Jochems, Xiaodi Liu, Yajun Cheng, Junfang Zhang, Salvatore Rudilosso, Agniete Kampaite, Olivia K.L. Hamilton, Rosalind Brown, Mark E. Bastin, Susana Muñoz Maniega, Iona Hamilton, Dominic Job, Fergus N. Doubal, Joanna M. Wardlaw

**Affiliations:** From the Row Fogo Centre for Research into Ageing and the Brain, Centre for Clinical Brain Sciences, and UK Dementia Research Institute (U.C., C.A.-R., D.J.G., W.H., R.L., M.D.C.V.H., S.J.W., M.S.S., M.T., F.M.C., A.C.C.J., A.K., O.K.L.H., R.B., M.E.B., S.M.M., I.H., D.J., F.N.D., J.M.W.), University of Edinburgh; Division of Neurology (X.L.), Department of Medicine, The University of Hong Kong; Department of Neurology (Y.C.), West China Hospital, Sichuan University, Chengdu, China; Department of Neurology (J.Z.), Shanghai General Hospital, Shanghai Jiao Tong University School of Medicine, China; Comprehensive Stroke Center (S.R.), Department of Neuroscience, Hospital Clinic, University of Barcelona and August Pi i Sunyer Biomedical Research Institute, Spain; and MRC/CSO Social and Public Health Sciences Unit (O.K.L.H.), School of Health and Wellbeing, University of Glasgow, United Kingdom.

## Abstract

**Background and Objectives:**

Factors associated with cerebral small vessel disease (SVD) progression, including incident infarcts, are unclear. We aimed to determine the frequency of incident infarcts over 1 year after minor stroke and their relation to baseline SVD burden, vascular risks, and recurrent stroke and cognitive outcomes.

**Methods:**

We recruited patients with lacunar or nondisabling cortical stroke. After diagnostic imaging, we repeated structural MRI at 3–6 monthly intervals for 12 months, visually assessing incident infarcts on diffusion-weighted imaging or FLAIR. We used logistic regression to determine associations of baseline vascular risks, SVD score, and index stroke subtype with subsequent incident infarcts. We assessed cognitive and functional outcomes at 1 year using Montreal Cognitive Assessment (MoCA) and modified Rankin scale (mRS), adjusting for baseline age, mRS, MoCA, premorbid intelligence, and SVD score.

**Results:**

We recruited 229 participants, mean age 65.9 (SD 11.1). Over half of all participants, 131 of 229 (57.2%) had had an index lacunar stroke. From baseline to 1-year MRI, we detected 117 incident infarcts in n = 57/229 (24.8%) participants. Incident infarcts were mainly of the small subcortical (86/117 [73.5%] in n = 38/57 [66.7%]) vs cortical infarct subtype (n = 19/57 [33.3%]). N = 39/57 participants had incident infarcts at 1 visit; 18 of 57 at 2 or more visits; and 19 of 57 participants had multiple infarcts at a single visit. Only 7 of 117 incident infarcts corresponded temporally to clinical stroke syndromes. The baseline SVD score was the strongest predictor of incident infarcts (adjusted odds ratio [OR] 1.87, 95% CI 1.39–2.58), while mean arterial pressure was not associated. All participants with incident infarcts were prescribed an antiplatelet or anticoagulant. Lower 1-year MoCA was associated with lower baseline MoCA (β 0.47, 95% CI 0.33–0.61), lower premorbid intelligence, and older age. Higher 1-year mRS was associated with higher baseline mRS only (OR 5.57 [3.52–9.10]). Neither outcome was associated with incident infarcts.

**Discussion:**

In the year after stroke in a population enriched for lacunar stroke, incident infarcts occurred in one-quarter and were associated with worse baseline SVD. Most incident infarcts detected on imaging did not correspond to clinical stroke/transient ischemic attack. Worse 1-year cognition and function were not associated with incident infarcts.

## Introduction

Cerebral small vessel disease (SVD) causes dementia, lacunar stroke, and intracerebral hemorrhage and is associated with other diverse clinical features including neuropsychiatric symptoms, impaired mobility, functional decline, and mortality.^1-5^ Structural SVD features visible on MRI include recent small subcortical infarcts and cortical microinfarcts.^6^ Our understanding of SVD mechanisms has advanced recently: SVD lesions can regress and progress^7,8^; evidence for interlesional interactions is growing^9,10^; and wide-ranging vascular impairments are linked to SVD including blood-brain barrier failure,^11,12^ impaired cerebrovascular reactivity,^13,14^ and increased intracranial pulsatility.^15,16^ Moreover, preclinical developments^17^ are establishing the pathophysiologic basis for SVD, and finally, potential treatments are starting to emerge.^18,19^

To build on these developments, we need better insight into the temporal nature of SVD lesion formation. Most studies have focused on chronic SVD lesion progression, using interscan intervals of 1 or more years,^7,20-24^ although a few have described SVD lesion evolution over days^25^ and weeks-months^26^ in relatively healthy populations. Increased use of MRI diffusion-weighted imaging (DWI) in SVD studies has highlighted that small DWI+ve lesions can occur without overt stroke symptoms, and these “incident infarcts” are a growing area of interest.^26^ It is not clear how frequently incident infarcts, visible on DWI or other sequences, of which many are clinically “covert,” occur following a stroke, and how these incident infarcts translate to clinical outcomes. Previous studies assessing incident infarcts after stroke,^27-31^ not SVD-specific, have recruited modest-sized populations with minor stroke (n < 100), with most attending 2 follow-up scans. Our understanding of incident infarct etiology, that is, how incident infarct subtype relates to index stroke subtype, for example, lacunar stroke, is limited. Although several studies, using Trial of ORG 10172 in Acute Stroke Treatment (TOAST) classification, have suggested that large-vessel index stroke subtypes carry the highest risk of developing incident infarcts,^27,31,32^ data on incident infarct subtypes, and thus index-incident etiologic associations, are rarely reported.^33^ Moreover, it is unclear how incident infarcts relate to vascular risk factors, guideline-based secondary prevention, and clinical outcomes.

We aimed to determine the frequency and timing of incident infarcts in the year after a minor lacunar or cortical stroke, to characterize incident infarct subtypes, and to assess how incident infarcts relate to baseline imaging features and vascular risks. Moreover, we aimed to determine whether incident infarcts were associated with recurrent stroke and 1-year cognitive and functional outcomes.

## Methods

The Mild Stroke Study 3 (MSS3) is a prospective observational study of patients with a nondisabling stroke who have had up to 4 clinical and imaging follow-up visits in 1 year. The full-study protocol is protocol published elsewhere,^34^ built on the foundations of previous longitudinal stroke studies at this center.^21,35^

We recruited adults presenting to Edinburgh/Lothian stroke services from 2018 to 2021 with mild ischemic stroke, defined as a modified Rankin scale (mRS) ≤2 at recruitment. The population was enriched for lacunar index stroke, with cortical stroke as controls, that is, we aimed for clinical lacunar stroke syndrome (50%) vs cortical ischemic stroke syndrome (50%), based on the clinical stroke syndrome presentation combined with review of imaging features. We excluded adults with MRI contraindications, severe cardiorespiratory comorbidities that would preclude lying flat, for example, advanced cardiac failure, and other neurologic conditions, for example, multiple sclerosis. Before recruitment, all patients received standard stroke investigations and management by stroke services according to guidelines. After diagnostic MRI/CT, we invited participants to attend baseline assessment <3 months after index stroke. We invited all participants to visits around 6 months and 1 year after the baseline assessment. We invited participants with index lacunar stroke or moderate-to-severe white matter hyperintensity (WMH) for 1 or 2 further MRI visits (eFigure 1). Where participants could not attend in person, we collected data by telephone, post, or from electronic records.

### Clinical Measurements

We recorded age, sex, and details of guideline-based investigation and management of acute stroke at baseline, as follows. We recorded vascular risk factors according to general practitioner coded diagnoses or hospital correspondence, including history of stroke or transient ischemic attack (TIA) before index stroke, diabetes, hypertension, hypercholesterolemia, atrial fibrillation, smoking status, ischemic heart disease (IHD), cardiac failure, valvular abnormalities, and patent foramen ovale (PFO) closure. We recorded results of electrocardiogram, echocardiography, and carotid imaging. We recorded prescribed secondary prevention medications at time of baseline visit. We did not record medications after baseline. We recorded sitting blood pressure (BP) and calculated mean arterial pressure (MAP; mm Hg) at each visit. We record the National Adult Reading Test (NART) as a measure of premorbid intelligence.^36^ At 1-year follow-up, we re-recorded vascular risk factors. We assessed functional and cognitive status at baseline and 1-year follow-up using mRS and Montreal Cognitive Assessment (MoCA), using different versions to avoid learning effects.^34^

We subtyped index stroke as previously,^21^ that is, clinical lacunar stroke syndrome and nonlacunar ischemic stroke syndromes (partial anterior circulation syndrome or posterior circulation syndrome) according to the Oxfordshire Community Stroke Project classification system,^37^ with recent infarct visible on diagnostic MRI or CT corresponding to the syndrome, or if no visible infarct, no other explanation for the stroke symptoms.

At each visit, we systematically enquired about stroke/TIA symptoms, before MRI. At 1-year follow-up, we defined recurrent stroke or TIA as a clinical stroke/TIA syndrome meeting diagnostic criteria, diagnosed either at stroke clinics or by study team consensus agreement.

### MRI Measurements

MRI methods are outlined in detail elsewhere.^34^ In brief, at index stroke diagnosis and pre-recruitment, all participants had a diagnostic scan, either at 3T (Siemens Prisma) MRI or 1.5T (General Electric Signa HDxt), or CT, with core structural sequences: 3D T1-weighted, T2-weighted, fluid-attenuated inversion recovery (FLAIR), susceptibility-weighted (susceptibility-weighted imaging/susceptibility-weighted angiography/gradient recalled echo), and diffusion tensor imaging. Within 3 months of index stroke, participants attended the baseline visit and had 3T MRI with the same core structural sequences, in addition to advanced neuroimaging.^34^ These structural sequences were repeated 3–9 months later in a subset (eFigure 1) with lacunar stroke or moderate-to-severe WMH to track serial lesion changes closely,^34^ and at 6 months and 1 year in all. All MRI from baseline to 1 year took place on the same 3T scanner which undergoes a continuous quality monitoring assurance programme.

We analyzed all images using STRIVE criteria.^6^ We visually rated index and incident infarcts and individual SVD features. We defined the index infarct, where present,^21^ as a recent infarct visible on diagnostic imaging that corresponded to the initial stroke syndrome, that is, pre-recruitment. We defined an incident infarct as any new infarct detected on baseline or later scans, that is, between index stroke diagnostic imaging and baseline or later, present on DWI or FLAIR and/or T2-weighted sequences, that was not present on previous scan/s. On baseline MRI, we constructed the summary SVD score, a composite score of visually rated WMH, perivascular spaces (PVS), microbleeds, and lacunes as previously described.^38^ An experienced team of individuals, supervised by a neuroradiologist (J.M.W.), visually assessed all index and incident infarcts on every diagnostic and study scan using a dedicated pro forma (eFigure 2), and classified the subtype and/or location of each lesion as small subcortical vs cortical.^39,40^ We analyzed images at all timepoints, including additional clinically indicated scans (eTable 1) using the same sequences during follow-up, for example, through the stroke service.

We analyzed WMH volumes as described previously, combining computational measures and careful visual checks.^34^ MRI raters were blinded to clinical information, except for final cross-checking.

### Statistical Analysis

We categorized each participant as having had a detectable “incident small subcortical infarct” vs “incident cortical infarct” vs “no incident infarct” between diagnostic imaging and 1-year follow-up. We defined vascular risk factors as present vs absent. For “any embolic source,” we summarized a composite variable containing ≥1 of the following: IHD, valvular heart disease, heart failure, atrial fibrillation, ≥70% stenosis of either internal carotid artery, and PFO closure.

We reported the mean (SD) or median (interquartile range [IQR]), and n (%) for descriptive data. We reported chi-squared values for univariate analyses of categorical, *t* tests for continuous, and Mann-Whitney *U* tests for ordinal variables. We used logistic regression to determine associations of incident infarcts with baseline age, smoking, prior stroke/TIA history, hypertension, hypercholesterolemia, proximal embolic source, summary SVD score, and index stroke subtype in a single model. We repeated adjustments in all models. We used ordinal logistic regression to determine whether 1-year mRS associated with incident infarcts and baseline age, prior stroke, SVD score, baseline mRS, baseline or NART. We used linear regression to determine whether 1-year MoCA associated with the same variables. All beta values were standardized. We performed the following sensitivity analyses where incident small subcortical infarct was the dependent variable: (1) we replaced incident small subcortical infarct with any incident infarct (i.e., combined incident small subcortical and cortical subtypes) as the dependent variable; (2) we replaced hypertension with baseline, 6 month, and 1 year MAP; and (3) we assessed incident infarcts detected at baseline only, to investigate effects of number of scans and attrition.

All analyses were performed using R version 4.2.2.^41^ We used packages finalfit^42^ and Modern Applied Statistics with S.^43^

### Standard Protocol Approvals, Registrations, and Patient Consents

Ethical approval for MSS3 was granted by the South East Scotland Research Ethics Committee (Ref 18/SS/0044), and all participants gave written informed consent.

### Data Availability

Anonymized data not published within this article can be made available by request from the corresponding author.

## Results

**Figure 1:**
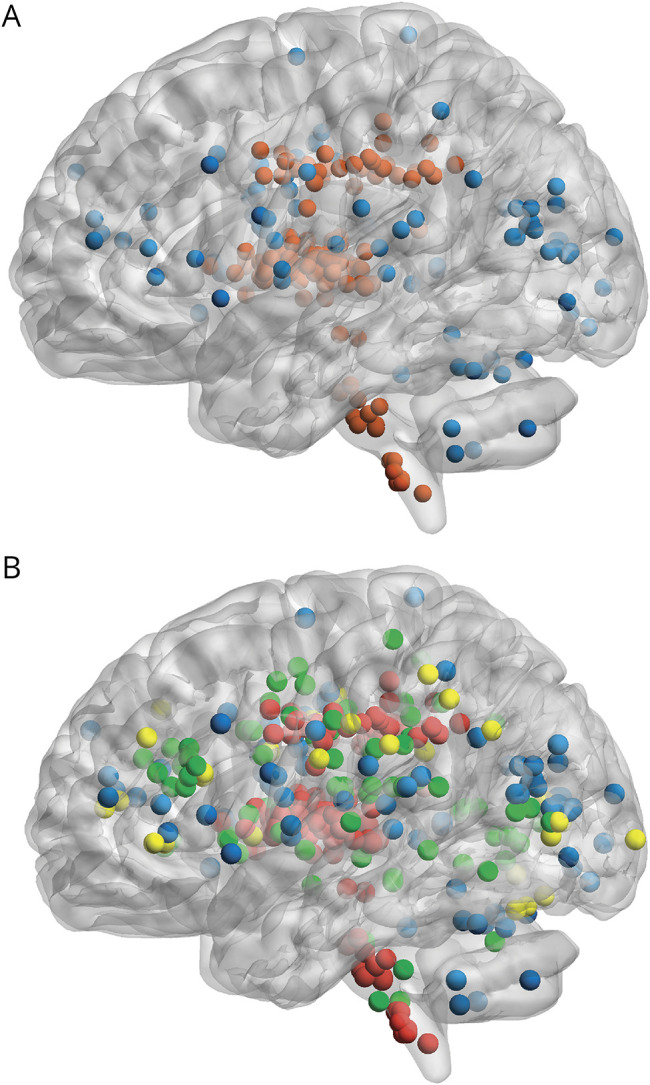
Index and Incident Infarct Locations (A) Index infarct locations across the study population showing population enriched for lacunar stroke. Red = small subcortical; blue = cortical. (B) Incident infarcts superimposed, n = 117, showing small subcortical infarcts accounting for the majority. Green = incident small subcortical infarct; yellow = incident cortical infarct; red = index small subcortical infarct; blue = index cortical infarct. Markers are a visual representation of incident infarct lesion location but do not represent lesion volume. Created using BrainNetViewer v1.7 and MRIcron, based on visual rating locations.

We recruited 229 participants of mean age 65.9 (SD 11.1) years. The index stroke subtype was defined as lacunar in 131 of 229 (57.2%). The median interval between index stroke onset and stroke service review was 2 (IQR 1–5) days. We describe baseline population characteristics in Table 1 and index stroke lesion locations in Figure 1 and eTable 2. Of 229 participants recruited, 215 of 229 (94%) had in-person or telephone follow-up and 203 of 229 (88.6%) attended 1-year follow-up MRI. Baseline differences between participants who did vs did not attend 1-year MRI are described in eTable 3 and reasons for loss to follow-up in eFigure 1. Participants who did not attend 1-year MRI were on average 3 years older, less likely to have had a previous stroke before the index stroke, more likely to have atrial fibrillation, and were more likely to have had an incident infarct at baseline (3/26 [11.5%] vs 13/203 [6.4%]), but there were no differences in baseline mRS or MoCA scores. Owing to coronavirus disease 2019 restrictions delaying 6-month and 1-year follow-ups, the mean interval between baseline and 6-month scan was 254.0 days (SD 36.9) and 1-year scan 437.7 days (SD 42.8).

**Table 1:** Baseline Population Characteristics According to Participants With vs Without Incident Infarcts Detected During Year of Follow-up After Stroke (n = 229)

	Any incident infarct (N = 57)	No incident infarct (N = 172)	Overall (N = 229)	*p* Value
Baseline age, y, mean (SD)	67.1 (11.0)	65.4 (11.2)	65.9 (11.1)	0.324
Female, n (%)	14 (24.6)	63 (36.6)	77 (33.6)	0.131
Index lacunar subtype, n (%)	37 (64.9)	94 (54.7)	131 (57.2)	0.229
Index cortical subtype, n (%)	20 (35.1)	78 (45.3)	98 (42.8)	
Prior history of TIA, n (%)	7 (12.3)	16 (9.3)	23 (10.0)	0.694
Prior history of stroke, n (%)	13 (22.8)	10 (5.8)	23 (10.0)	<0.001
Prior history of stroke/TIA, n (%)	16 (28.1)	20 (11.6)	36 (15.7)	0.006
Diabetes, n (%)	18 (31.6)	32 (18.6)	50 (21.8)	0.061
Hypertension, n (%)	42 (73.7)	115 (66.9)	157 (68.6)	0.425
MAP, mm Hg, mean (SD)	108 (12.5)	105 (13.2)	106 (13.1)	0.099
Hypercholesterolemia, n (%)	43 (75.4)	128 (74.4)	171 (74.7)	>1
Smoker: current or ex <1 y, n (%)	13 (22.8)	28 (16.3)	41 (17.9)	0.319
Smoker: never or ex >1 y, n (%)	42 (73.7)	142 (82.6)	184 (80.3)	
Atrial fibrillation, n (%)	4 (7.0)	17 (9.9)	21 (9.2)	0.7
Ischemic heart disease, n (%)	9 (15.8)	15 (8.7)	24 (10.5)	0.207
Heart failure, n (%)	0 (0)	3 (1.7)	3 (1.3)	0.74
Valvular heart disease, n (%)	3 (5.3)	7 (4.1)	10 (4.4)	0.993
PFO closure, n (%)^a^	1 (1.8)	8 (4.7)	9 (3.9)	0.56
Right ICA^b^ <50% or not quantified or not assessed, n (%)	56 (98.2)	167 (97.1)	223 (97.4)	0.573
Right ICA 50%–69%, n (%)	0 (0)	3 (1.7)	3 (1.3)	
Right ICA ≥70%, n (%)	1 (1.8)	2 (1.2)	3 (1.3)	
Left ICA <50% or not quantified or not assessed, n (%)	54 (94.7)	166 (96.5)	220 (96.1)	0.732
Left ICA 50%–69%, n (%)	2 (3.5)	3 (1.7)	5 (2.2)	
Left ICA ≥70%, n (%)	1 (1.8)	3 (1.7)	4 (1.7)	
Any potential proximal embolic source, n (%)^c^	17 (29.8)	43 (25.0)	58 (25.3)	0.586
Antiplatelet, n (%)	51 (89.5)	153 (89.0)	204 (89.1)	>0.99
Anticoagulant, n (%)	7 (12.3)	19 (11.0)	26 (11.4)	0.98
Antihypertensive/s, n (%)	40 (70.2)	111 (64.5)	151 (65.9)	0.53
Lipid-lowering agent, n (%)	53 (93.0)	158 (91.9)	211 (92.1)	>0.99
Baseline SVD score, mean (SD)	2.54 (1.21)	1.57 (1.29)	1.82 (1.34)	<0.001
Baseline SVD score, median (Q1, Q3)	3 (2, 4)	2 (0, 3)	2 (1, 3)	<0.001

Abbreviations: ICA = internal carotid artery; MAP = mean arterial pressure; PFO = patent foramen ovale; SVD = small vessel disease; TIA = transient ischemic attack.

a Refers to PFO closure between index stroke and 1-year follow-up.

b Refers to ICA stenosis at index stroke.

c Refers to ≥1 of atrial fibrillation (n = 19), ≥70% North American Symptomatic Carotid Endarterectomy Trial stenosis of either internal carotid artery, PFO closure, ischemic heart disease, valvular heart disease, heart failure.

### Incident Infarct Frequency, Timing, and Characteristics

The 229 participants attended 1,021 MRI scans, including index stroke diagnostic imaging and additional clinically indicated scans, with all participants having had diagnostic imaging performed by clinical stroke services at presentation. The mean interval between index stroke symptoms and baseline study MRI was 51.5 (SD 21.9) days. From baseline to 1-year MRI, we detected 117 incident infarcts in n = 57/229 (24.8%) participants at 79 separate visits. Most incident infarcts were of the small subcortical subtype: 86 of 117 (73.5%) total incident infarcts in n = 38/57 (66%) participants. eFigure 3 shows incident infarct subtype according to index stroke subtype. N = 39 participants had incident infarcts detected at 1 visit; n = 14 at 2 visits; n = 3 at 3 visits, and n = 1 at 4 visits. Nineteen participants had ≥2 incident infarcts detected at a single visit, and the maximum number of infarcts detected in a single participant at a single visit was 5. Assessing 117 incident infarcts on different sequences, 105 of 117 were hyperintense on DWI; 103 of 117 were visible on FLAIR sequences; and 92 of 117 were visible on both sequences. Eleven lesions were only visible on FLAIR; 13 lesions were only visible on DWI; and 1 lesion was detected on a clinically indicated CT before loss to MRI follow-up. Clinically overt stroke or TIA symptoms corresponded clinically and temporally to incident infarcts in 6 of 57 participants (10.5%), 4 of 57 to lacunar and 2 of 57 to cortical stroke syndromes.

**Figure 2:**
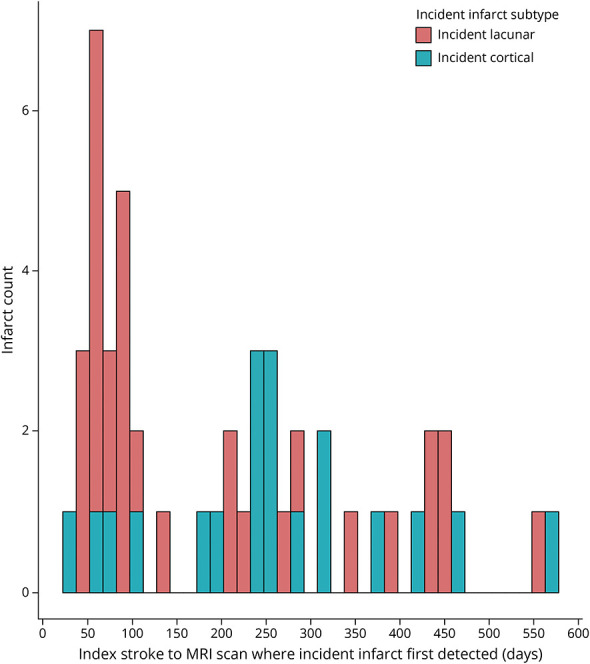
Distribution Timeline for Detection of All Incident Infarcts at All Visits (n = 117), Including Multiple Incident Infarcts Per Participant

The distribution timeline for all detected incident infarcts is presented in Figure 2. The first incident infarct per participant was detected at median 83 days (IQR 59–236) after index stroke, that is, more often at the baseline visit that occurred within 3 months of index stroke than later, for example, 49 incident infarcts were detected at median 2.5 months vs 20 infarcts detected at 12 months. Small subcortical infarcts were first detected earlier (median 71 days [IQR 51.5–109]) than cortical infarcts (median 235 days [IQR 83–316]).

Differences between participants with vs without any incident infarct are outlined in Table 1 and differences for participants with incident small subcortical vs cortical infarcts in eTable 4. The study team assessed all participants with small subcortical infarcts for evidence of ongoing embolic risk and apart from 2 participants with a history of atrial fibrillation, who were already anticoagulated, we did not identify any other active embolic sources. eTable 5 presents individual components of the summary SVD score, that is, baseline WMH, lacunes, microbleeds, PVS, in participants with vs without incident infarcts. The majority of incident small subcortical infarcts were located in the centrum semiovale (21.4%), optic radiation (10.3%), and juxta-cortical regions (8.5%), while most incident cortical infarcts were middle cerebral artery (9.4%), posterior borderzone (6.8%), and posterior cerebral artery territories (3.4%). Incident infarct locations are outlined in full in Figure 1 and Table 2.

**Table 2:** Locations of 117 Incident Infarcts in 57 of 229 Participants, Ordered by Decreasing Frequency

	Incident infarct total (N = 117)
Incident small subcortical infarct locations, n (%)	
Centrum semiovale	25 (21.4)
Optic radiation	12 (10.3)
Juxta-cortical	10 (8.5)
Internal border zone	7 (6.0)
Thalamus	7 (6.0)
Lentiform nucleus	6 (5.1)
Pons	6 (5.1)
Internal capsule	3 (2.6)
Anterior frontal	3 (2.6)
Cerebellum	2 (1.7)
External capsule	1 (0.9)
Subcortical	1 (0.9)
Anterior temporal	1 (0.9)
Splenium of corpus callosum	1 (0.9)
Other	1 (0.9)
Incident cortical infarct locations, n (%)	
Small middle cerebral artery	11 (9.4)
Posterior borderzone	8 (6.8)
<Half posterior cerebral artery territory	4 (3.4)
Anterior borderzone	3 (2.6)
<Half anterior cerebral artery territory	2 (1.7)
<Half hemisphere	1 (0.9)
Posterior half peripheral middle cerebral artery territory	1 (0.9)
Whole peripheral + lateral basal ganglia	1 (0.9)

Of 97 incident infarcts detected between median 2.5 and 6 months after stroke, 4 participants with 7 incident infarcts had loss to imaging follow-up by 1 year due to participant inability to attend the scan. Of the remaining 90 infarcts followed up to 1 year, 78 of 90 (86.6%) remained visible and 12 of 90 (13.3%) were no longer visible. The median interval between baseline scan and 1-year scan in patients with incident infarcts which remained visible was 437 days vs 415 days in patients whose incident infarcts were not visible.

**Figure 3:**
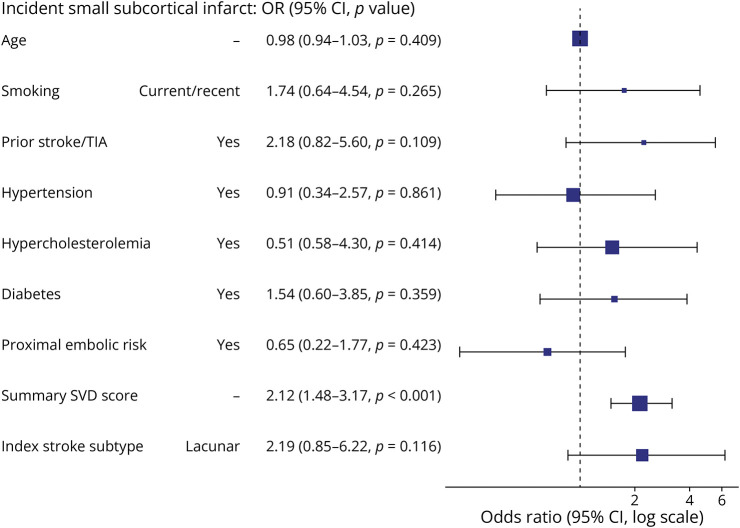
Baseline Factors Associated With Incident Small Subcortical Infarct During 1-Year Follow-Up OR = odds ratio; SVD = small vessel disease; TIA = transient ischemic attack.

In univariate analyses, incident infarct subtype was associated with index stroke subtype, that is, individuals who had had an index lacunar stroke diagnosis were more likely to develop incident small subcortical infarcts than cortical infarcts, χ^2^ = 11.014 (*p* = 0.004) (eFigure 3). In multivariable analysis, the baseline summary SVD score was the strongest predictor of participants having detectable incident small subcortical infarcts: odds ratio (OR) 2.12 (95% CI 1.48–3.17, *p* < 0.001); and to a lesser extent, index lacunar stroke subtype and prior stroke/TIA history (Table 3 and Figure 3). In sensitivity analysis, the baseline summary SVD score remained the strongest predictor of participants developing any incident infarct, regardless of subtype (OR 1.87, 95% CI 1.39–2.58) (eTable 6). In sensitivity analyses of incident infarcts detected at the baseline visit only (n = 19 with incident infarcts at baseline vs n = 210 without), the summary SVD score remained strongly associated with incident infarcts (eFigure 4).

**Table 3:** Baseline Factors Associated With Incident Infarcts

Dependent: incident small subcortical infarct	No incident small subcortical infarct	Incident small subcortical infarct	OR (univariable)	OR (multivariable)
Age, y, mean (SD)	66.0 (11.1)	65.1 (11.6)	0.99 (0.96–1.02, *p* = 0.653)	0.98 (0.94–1.03, *p* = 0.409)
Smoking: current/ex <1y	30 (15.9)	11 (30.6)	2.33 (1.01–5.16, *p* = 0.040)	1.74 (0.64–4.54, *p* = 0.265)
Prior stroke/TIA	24 (12.6)	12 (31.6)	3.21 (1.40–7.13, *p* = 0.005)	2.18 (0.82–5.60, *p* = 0.109)
Hypertension	128 (67.0)	29 (76.3)	1.59 (0.73–3.74, *p* = 0.262)	0.91 (0.34–2.57, *p* = 0.861)
Hypercholesterolemia	141 (73.8)	30 (78.9)	1.33 (0.60–3.28, *p* = 0.508)	1.51 (0.58–4.30, *p* = 0.414)
Diabetes	38 (19.9)	12 (31.6)	1.86 (0.84–3.96, *p* = 0.115)	1.54 (0.60–3.85, *p* = 0.359)
Proximal embolic risk	52 (27.2)	8 (21.1)	0.71 (0.29–1.59, *p* = 0.431)	0.65 (0.22–1.77, *p* = 0.423)
Summary SVD score, mean (SD)	1.6 (1.3)	2.8 (1.1)	2.03 (1.51–2.81, *p* < 0.001)	2.12 (1.48–3.17, *p* < 0.001)
Index stroke subtype: Lacunar	101 (52.9)	30 (78.9)	3.34 (1.52–8.16, *p* = 0.004)	2.19 (0.85–6.22, *p* = 0.116)

Abbreviations: SVD = small vessel disease; TIA = transient ischemic attack.

### Secondary Prevention, Stroke Recurrence, and Cognitive Outcomes

Participants' prescribed secondary prevention medications according to guidelines at the time of the baseline visit, that is, the date of the first study scan, were as follows: 204 of 229 (89.0%) participants were prescribed antiplatelets, 26 of 229 (11.3%) anticoagulants, 211 of 229 (92.1%) lipid-lowering therapy, and 151 of 229 (65.9%) ≥1 antihypertensive agent. Secondary prevention prescribed at baseline visit is in Table 1. Of the antiplatelet therapy group, 94.1% (192/204) were prescribed clopidogrel only, 2.9% (6/204) were prescribed aspirin only, and 2.9% (6/204) were prescribed dual antiplatelet therapy. Regarding antihypertensive agents, 74 of 229 (32.4%) were prescribed a single antihypertensive, 56 of 229 (24.4%) 2 antihypertensives, 17 of 229 (7.4%) 3 antihypertensives, 3 of 229 (1.3%) 4 antihypertensives, and 1 of 229 (0.4%) 7 antihypertensives. Seventy-eight of 229 (34.1%) participants were not prescribed any antihypertensive on the date of the baseline visit.

Of the 57 participants with incident infarcts, 53 of 57 (92.9%) were prescribed lipid-lowering treatment, 57 of 57 were prescribed an antiplatelet or anticoagulant, and 40 of 57 (70.1%) were prescribed at least 1 antihypertensive at the time of their baseline visit. Of the 17 of 57 (29.8%) who were not prescribed any antihypertensive on the date of the baseline visit, baseline BP was >140/90 mm Hg in 14 of 17 and >130/80 mm Hg in 17 of 17 participants.

There was minimal change in vascular risk factor prevalence and smoking status between baseline and 1-year follow-up (eFigure 5, A and B). At 1 year, BP was reported as poorly controlled in 16 of 219 (7.3%) (eFigure 5C). Sensitivity analyses including MAP instead of hypertension diagnosis at baseline, 6 months, and 1 year in the incident infarct model did not show any association between MAP and incident small subcortical infarcts (eFigure 6).

At 1 year, 18 of 229 (7.8%) participants had had 23 episodes of recurrent clinical stroke/TIA (13 ischemic stroke; 10 TIA). The median interval between index stroke and first clinical stroke/TIA recurrence was 196 (IQR 264–315.5) days. Of the 57 participants with incident infarcts on MRI, 6 of 57 (10.5%) participants had 7 infarcts that corresponded clinically and temporally to explained acute stroke symptoms. Five of these strokes were diagnosed by clinical stroke services and 2 by the study team. A further 2 of 57 participants with incident infarcts developed clinical stroke/TIA recurrence that was separated in time and space from their incident infarct, that is, 2 participants did not have any acute lesions detected at MRI scans that were performed as part of acute stroke imaging but did have incident infarcts detected at distant study visits that did not correspond to recurrent stroke symptoms.

Two participants had acute stroke with incident infarcts on CT remote from study MRI but did not attend MRI follow-up and are not included in the present MRI-based analysis.

At 1 year, higher 1-year mRS was associated with higher baseline mRS only (OR 5.57 [3.52–9.10]) (Table 4). Lower 1-year MoCA scores were associated with lower baseline MoCA (β 0.47, 95% CI 0.33–0.61), lower premorbid intelligence (β 0.07, 95% CI 0.02–0.12), and older age (β −0.06, 95% CI −0.10 to −0.02) (eTable 7). Neither mRS nor MoCA was associated with incident infarcts.

**Table 4:** Ordinal Regression of Factors Associated With Odds of Developing Increasingly Impaired Modified Rankin Scale at 1 Year, Adjusted

	OR (95% CI)
Baseline age	1.01 (0.98–1.04)
Prior stroke history	1.68 (0.64–4.40)
Baseline SVD score	1.14 (0.90–1.44)
Baseline mRS	5.57 (3.52–9.10)
Baseline MoCA	1.00 (0.91–1.10)
NART	0.99 (0.95–1.02)
Any incident infarct	0.95 (0.48–1.84)

Abbreviations: MoCA = Montreal Cognitive Assessment; mRS = modified Rankin scale; NART = National Adult Reading Test; OR = odds ratio; SVD = small vessel disease.

## Discussion

In a population enriched for lacunar stroke, incident infarcts in the year after stroke were detectable on serial brain MRI in 25%, were mostly subcortical, and were associated with worse baseline SVD. These findings support that having an existing high SVD burden generates worsening SVD and that etiologic stroke subtypes hold true, that is, incident SVD-related infarcts are propagated by SVD that progresses after an index lacunar stroke event. Moreover, our findings show that conventional secondary stroke prevention seems not to be very effective at preventing incident infarcts in patients with SVD-related strokes. These findings should be interpreted considering that the population was enriched for index lacunar stroke (57% in this cohort vs 25% in the general ischemic stroke population).

Incident infarcts, particularly small subcortical infarcts, were first detected in the early rather than later months after stroke (median 83 days), and overall median 2.5 months after stroke, that is, outside the time of highest stroke recurrence risk for large artery and cardioembolic stroke.^31^ Incident infarcts occurred despite guideline-based secondary stroke prevention in the majority, highlighting a need for more effective treatments for lacunar stroke and for SVD more generally.

In most individuals, incident infarcts were clinically covert according to existing diagnostic criteria for stroke (51/57 [89.4%]) and were not associated with 1-year mRS or MoCA.

Compared with previous studies, our study was enriched for lacunar stroke so better reflects the natural history of SVD lesion progression in a lacunar stroke population. A previous study of 143 stroke/TIA patients (44% undetermined etiology) with follow-up MRI at 30 days detected incident infarcts in 10% (14/143).^29^ In 50 unsubtyped stroke/TIA patients with follow-up MRI at 7 and 30 days poststroke, incident infarcts were detected in 18% (9/50) at 7 days and 22% (11/50) at 30 days.^30^ A smaller study of 34 stroke patients with risk factors for large artery atherosclerosis followed up with MRI monthly for 1–9 months and detected infarcts in 11% (4/34).^28^ No previous studies evaluated summary SVD scores or incident infarct subtype. Our findings of a high rate of recurrent small subcortical infarcts contrast with previous work suggesting that early clinical stroke recurrence mainly occurs in individuals with initial large vessel etiology (based on TOAST classification).^31^ Our results shift the narrative on predominant etiologies of stroke disease recurrence in a number of ways: populations enriched for lacunar stroke are different to the wider stroke population, imaging recurrence of infarcts exceeds clinical recurrence and is sensitive to the major contribution of SVD, and including imaging in diagnostic classifications provides better insight into the underlying mechanisms of index stroke and thus mechanisms of stroke recurrence. Our findings support the need for clinicians to pay very close attention to acute small subcortical infarcts reported on diagnostic imaging when considering future infarct recurrence risk.

Most incident infarcts in our study were clinically covert (only 10.5% corresponded to stroke symptoms) vs previous studies (24%–57%).^28-30^ This may be explained by the high incidence of small subcortical infarcts in our population, which may have been too small or located in less eloquent brain regions to result in clinical strokes, for example, not affecting narrow motor and sensory tracts.^44^ Moreover, the mechanisms underlying SVD progression affect more than just the vessel lumen and can damage brain tissue more slowly, that is, not just over seconds and minutes, but also over days, weeks, months, and years, so a less abrupt symptom onset than for large-vessel strokes is to be expected at least some of the time. It is possible therefore that progressing SVD results in chronic and progressive atypical symptoms, or no symptoms, punctuated by acute episodes that match textbook descriptions of clinical stroke syndromes. Finally, clinicians' current understanding of lesion location and symptom interpretation has arisen from patterns observed in older imaging and pathologic studies which have limitations for observing real-time symptom-lesion changes. Our findings highlight that clinical recurrences of conventional stroke syndromes are simply the “tip of the iceberg” compared with MRI recurrences of infarcts and highlight the need for further research into atypical symptoms.

Our study had a higher frequency of incident infarcts (25%) than a previous study assessing nonstroke patients at high risk for SVD progression: 16.6% (9/54; 10 serial scans/10-month follow-up).^26^ However, this study assessed DWI-hyperintense lesions only, and the incident infarcts were mostly cortical, likely reflecting different population characteristics. In a longer study of older adults over 5 years, incident infarcts occurred in only 20.8% (545/2,612).^45^ The higher frequency of incident infarcts over a shorter timeframe in our study reflects the unique nature of acute lacunar stroke populations.

Our data add weight to existing studies showing that SVD increases future stroke risk in patients with a history of any stroke^46^ and in individuals who have not yet had a stroke.^4^ Our findings give a plausible explanation for why this may be happening, that is, SVD is a major contributor but also suggests that our understanding may only have been the tip of the iceberg because it is now clearer that many incident infarcts do not cause stroke symptoms. Despite being mostly covert or apparently “silent,” incident infarcts have serious clinical implications because they are linked with increased risk of dementia and stroke.^47^

This study found that SVD is the leading contributor to incident subcortical infarcts. The strong links between index lacunar, summary SVD score, and the development of incident small subcortical infarcts in this study supports that etiology holds true^48^ and that SVD generates worsening SVD in some, but not all. Moreover, this study confirms that preventing SVD progression is not as simple as treating vascular risk factors, for example, BP, according to current secondary prevention guidelines, as noted for covert SVD,^49^ and incident infarcts occurred even in individuals with a history of prior stroke/TIA who were already taking secondary prevention. Current best evidence on management of covert SVD is outlined elsewhere,^49^ and promising SVD treatments are emerging.^18^

The study has limitations. The 51.5-day mean interval between index stroke and baseline visit means we may have missed early incident small infarcts in the cortical group because the risk for recurrent large-vessel stroke is highest in the early period after stroke. We may have not detected infarcts and strokes/TIAs that appeared and disappeared between visits. However, our visits were more frequent than most previous stroke imaging studies, and the majority of incident infarcts detected by 6-month follow-up remained visible around 1 year (86.6%). Not all participants attended an extra scan (eFigure 1) in which 17 of 117 incident infarcts were detected. However, 15 of 17 infarcts remained visible at later visits so most would have been detected. In addition, we performed a sensitivity analysis assessing incident infarcts detected at the baseline visit only, and the summary SVD score remained strongly associated with incident infarcts. The incident infarcts are only part of the full SVD picture, and future analyses should include other SVD features. We did not detect associations between incident infarcts and cognitive and functional outcomes at 1-year follow-up. Such associations may become apparent in future analyses of this population at 2-, 3-, and 4-year follow-up because some vascular risk factors require longer follow-up to determine clinically relevant effects. Our assessment of secondary prevention medications only refers to medications prescribed on the date of the baseline visit, whereas follow-up medication data would have been useful to determine whether medication changes may have related to incident infarcts, although we did assess MAP at each timepoint.

Although all participants with incident infarcts were taking antiplatelet and lipid-lowering therapies, up to 30% were not prescribed antihypertensives at least by the baseline visit, and patient-reported BP control in the year after stroke was suboptimal, consistent with widely reported data^50^; however, BP control in participants with incident infarcts was no worse than participants without.

The study strengths include detailed serial imaging, with visual ratings on >1,000 scans by an experienced team. Our use of visual ratings allows immediate clinical translation. We scanned participants frequently, increasing the likelihood of capturing dynamic lesion changes. We included incident infarcts on FLAIR and on DWI, increasing the chances of detecting nonacute lesions. We had high scan retention rates at 6 months (89.0%) and 1 year (88.6%) (eFigure 1).

This study's findings support the need for better treatments of lacunar stroke and SVD more generally. We also need to focus on better ways to detect SVD progression in individuals who have not yet had a clinical stroke because our findings confirm that the majority of individuals with incident small subcortical infarcts do not present with stroke symptoms according to current accepted definitions. We need to determine whether there are other symptoms apart from classic stroke syndromes, for example, neuropsychiatric, gait, and subtle cognitive symptoms, that correspond to incident infarct development. Identifying populations with SVD will be increasingly aided by data linkage of routine imaging, and for those individuals who have not had imaging, progressing research on symptom and biomarker risk profiles. The main focus should be on early identification, years before stroke or dementia occur. Further work will allow us to longitudinally track lesions and clinical course contemporaneously to uncover targetable mechanisms at targetable timepoints, assessing different stages and rates of SVD progression, including early warning symptoms, biomarkers, and subvisible changes and advanced neuroimaging features of small vessel dysfunction.

## Appendix Authors

**Table:**

Name	Location	Contribution
Una Clancy, PhD	Centre for Clinical Brain Sciences and the UK Dementia Research Institute at the University of Edinburgh	Drafting/revision of the manuscript for content, including medical writing for content; major role in the acquisition of data; analysis or interpretation of data
Carmen Arteaga-Reyes, PhD	Centre for Clinical Brain Sciences and the UK Dementia Research Institute at the University of Edinburgh	Drafting/revision of the manuscript for content, including medical writing for content; major role in the acquisition of data
Daniela Jaime Garcia, MSc	Centre for Clinical Brain Sciences and the UK Dementia Research Institute at the University of Edinburgh	Drafting/revision of the manuscript for content, including medical writing for content; major role in the acquisition of data
Will Hewins, MSc	Centre for Clinical Brain Sciences and the UK Dementia Research Institute at the University of Edinburgh	Major role in the acquisition of data
Rachel Locherty, MSc	Centre for Clinical Brain Sciences and the UK Dementia Research Institute at the University of Edinburgh	Drafting/revision of the manuscript for content, including medical writing for content; major role in the acquisition of data
Maria Del C. Valdés Hernández, PhD	Centre for Clinical Brain Sciences and the UK Dementia Research Institute at the University of Edinburgh	Drafting/revision of the manuscript for content, including medical writing for content; major role in the acquisition of data
Stewart J. Wiseman, PhD	Centre for Clinical Brain Sciences and the UK Dementia Research Institute at the University of Edinburgh	Major role in the acquisition of data; analysis or interpretation of data
Michael S. Stringer, PhD	Centre for Clinical Brain Sciences and the UK Dementia Research Institute at the University of Edinburgh	Major role in the acquisition of data
Michael Thrippleton, PhD	Centre for Clinical Brain Sciences and the UK Dementia Research Institute at the University of Edinburgh	Major role in the acquisition of data
Francesca M. Chappell, PhD	Centre for Clinical Brain Sciences and the UK Dementia Research Institute at the University of Edinburgh	Drafting/revision of the manuscript for content, including medical writing for content; major role in the acquisition of data; analysis or interpretation of data
Angela C.C. Jochems, PhD	Centre for Clinical Brain Sciences and the UK Dementia Research Institute at the University of Edinburgh	Drafting/revision of the manuscript for content, including medical writing for content; major role in the acquisition of data
Xiaodi Liu, PhD	Division of Neurology, Department of Medicine, The University of Hong Kong	Drafting/revision of the manuscript for content, including medical writing for content; major role in the acquisition of data
Yajun Cheng, PhD	Department of Neurology, West China Hospital, Sichuan University, Chengdu, China	Drafting/revision of the manuscript for content, including medical writing for content; major role in the acquisition of data
Junfang Zhang, PhD	Department of Neurology, Shanghai General Hospital, Shanghai Jiao Tong University School of Medicine, China	Drafting/revision of the manuscript for content, including medical writing for content; major role in the acquisition of data
Salvatore Rudilosso, PhD	Comprehensive Stroke Center, Department of Neuroscience, Hospital Clinic, University of Barcelona and August Pi i Sunyer Biomedical Research Institute, Spain	Drafting/revision of the manuscript for content, including medical writing for content; major role in the acquisition of data
Agniete Kampaite, BSc	Centre for Clinical Brain Sciences and the UK Dementia Research Institute at the University of Edinburgh	Major role in the acquisition of data
Olivia K.L. Hamilton, PhD	Centre for Clinical Brain Sciences and the UK Dementia Research Institute at the University of Edinburgh; MRC/CSO Social and Public Health Sciences Unit, School of Health and Wellbeing, University of Glasgow, United Kingdom	Drafting/revision of the manuscript for content, including medical writing for content; major role in the acquisition of data
Rosalind Brown, PhD	Centre for Clinical Brain Sciences and the UK Dementia Research Institute at the University of Edinburgh	Major role in the acquisition of data
Mark E. Bastin, DPhil	Centre for Clinical Brain Sciences and the UK Dementia Research Institute at the University of Edinburgh	Drafting/revision of the manuscript for content, including medical writing for content; major role in the acquisition of data
Susana Muñoz Maniega, PhD	Centre for Clinical Brain Sciences and the UK Dementia Research Institute at the University of Edinburgh	Drafting/revision of the manuscript for content, including medical writing for content; major role in the acquisition of data
Iona Hamilton, BSc	Centre for Clinical Brain Sciences and the UK Dementia Research Institute at the University of Edinburgh	Major role in the acquisition of data
Dominic Job, PhD	Centre for Clinical Brain Sciences and the UK Dementia Research Institute at the University of Edinburgh	Major role in the acquisition of data
Fergus N. Doubal, PhD	Centre for Clinical Brain Sciences and the UK Dementia Research Institute at the University of Edinburgh	Drafting/revision of the manuscript for content, including medical writing for content; major role in the acquisition of data; study concept or design; analysis or interpretation of data
Joanna M. Wardlaw, MD	Centre for Clinical Brain Sciences and the UK Dementia Research Institute at the University of Edinburgh	Drafting/revision of the manuscript for content, including medical writing for content; major role in the acquisition of data; study concept or design; analysis or interpretation of data

## Glossary

BP: blood pressure
DWI: diffusion-weighted imaging
FLAIR: fluid-attenuated inversion recovery
IHD: ischemic heart disease
IQR: interquartile range
MAP: mean arterial pressure
MoCA: Montreal Cognitive Assessment
mRS: modified Rankin scale
MSS3: Mild Stroke Study 3
NART: National Adult Reading Test
OR: odds ratio
PFO: patent foramen ovale
PVS: perivascular spaces
SVD: small vessel disease
TIA: transient ischemic attack
TOAST: Trial of ORG 10172 in Acute Stroke Treatment
WMH: white matter hyperintensity
